# Practice effects on dual-task order coordination and its sequential adjustment

**DOI:** 10.3758/s13423-024-02476-6

**Published:** 2024-02-26

**Authors:** Tilo Strobach

**Affiliations:** 1 Department of Psychology, Medical School Hamburg, Am Kaiserkai 1, 20457 Hamburg, Germany; 2 ICAN Institute for Cognitive and Affective Neuroscience, Medical School Hamburg, Am Kaiserkai 1, 20457 Hamburg, Germany

**Keywords:** Dual tasks, Dual-task practice, Task order, Task-order control, Cognitive plasticity

## Abstract

When the performance of two tasks overlaps in time, performance impairments in one or both tasks are common. Various theoretical explanations for how component tasks are controlled in dual-task situations have been advanced. However, less attention has been paid to the issue of how two temporally overlapping tasks are appropriately coordinated in terms of their order. The current study focuses on two specific aspects of this task-order coordination: (1) the potential effects of practice on task-order coordination performance and (2) its relationships with cognitive meta-control mechanisms that adjust this coordination. These aspects were investigated in a visual-auditory dual-task combination with randomly changing task orders across trials after four sessions of dual-task practice (*N* = 24) and single-task practice (*N* = 24). The results demonstrated that task-order coordination improves during dual-task practice, and in contrast to the effects of single-task practice. Practice, on the other hand, did not show substantial evidence of an effect on the adjustment of task-order coordination. This practice-related dissociation is consistent with the assumption that (1) task-order coordination and (2) its sequential adjustment are separable sets of processes.

**Supplementary information:**

The online version contains supplementary material available at 10.3758/s13423-024-02476-6.

## Introduction

When the performance of two tasks is temporally overlapping, performance decrements usually occur in one or both of the tasks. The most widely used experimental paradigm to study such dual-task performance costs is the “psychological refractory period” (PRP) paradigm (Telford, 1931; Welford, 1952). In the PRP paradigm, a first task (T1) and a second task (T2) are presented with varying intervals (i.e., stimulus onset asynchronies; SOAs) between the tasks, and participants are instructed to respond to both tasks with priority on T1. The typical finding is that reaction times to T2 (RT2) increase with decreasing SOAs; this RT2 pattern is referred to as the “PRP effect.” This PRP effect has been attributed to capacity limitations on T1 and T2 response selection stages that usually process only one task at a time and, thus, result in the serial processing of both tasks (Pashler, 1994; Schubert, 1999).

Various theoretical explanations have been advanced for how capacity limitations in component tasks are controlled in the PRP paradigm (e.g., Koch et al., 2018; Pashler, 1994). However, considerably less attention has been paid to the issue that two temporally overlapping tasks, if they are to be performed correctly and efficiently, must, first of all, be scheduled appropriately in terms of their processing and response order (e.g., de Jong, 1995; Luria & Meiran, 2003; Szameitat et al., 2006). The present study focuses on two particular aspects of this task-order coordination (TOC): (1) the potential effects of practice on TOC, and (2) the adjustment of TOC (i.e., coordination adjustment; CA).

### Task-order coordination and sequential adjustment of task-order coordination

Dual-task situations require some type of task control to actively coordinate the processing streams of the two component tasks (e.g., Strobach, 2020). For this coordination, participants need, for instance, information about which two component tasks they have to perform in a dual-task situation. Evidence for this type of task coordination comes from PRP studies using the task-pair switching logic (e.g., Hirsch et al., 2017, 2018, 2021). In this logic, three tasks (Tasks A, B, and C) are combined into two task pairs. T1 is constant and T2 varies across task pairs (e.g., Task-Pair 1 with Task C as T1 and Task A as T2; Task-Pair 2 with Task C as T1 and Task B as T2) or vice versa, and the task-pair sequence is varied on a trial-by-trial basis within experimental blocks. With this logic, performance in T1 and T2 is typically impaired in task-pair switch trials (e.g., Task-Pair 1 → Task-Pair 2) compared with task-pair repetition trials (e.g., Task-Pair 2 → Task-Pair 2), resulting in task-pair switch costs. Since there is a task switch between T2 in Trial *N* − 1 and T1 in Trial *N* in task-pair switch trials and task-pair repetition trials, task-pair switch costs and thus task coordination are not attributable to “local” switching between the individual component tasks across trials.

Another important aspect of the PRP paradigm is that two punctate, nonideomotor compatible tasks^1^ need to be temporally scheduled (i.e., brought into a specific order) for adequate performance. This is particularly relevant when the order of the component tasks randomly varies between trials and when task stimulus presentation order and response execution order should be consistent. Recently, increased RTs in both component tasks were consistently observed when switching (vs. repeating) the task order from the previous PRP Trial *N* − 1 to the current PRP Trial *N* (i.e., *order switch costs*; e.g., Kübler et al., 2022a, 2022b; Luria & Meiran, 2003, 2006; Strobach et al., 2021a; Szameitat et al., 2006), indicating active TOC. Similar to the task-pair switching logic, order switch costs could not be reduced to task switches at the “local” component-task level. This is so because task-order switches are connected with repetitions at the component-task level across trials (e.g., task orders B[A → A]B; see Table 1), whereas task-order repetitions have switches at the component-task level (e.g., task orders A[B → A]B). If “local” switching would explain the order switch costs exclusively, then task-order switches should rather reduce than increase these costs.

As reviewed by Strobach (in press), order switch costs were shown to be modulated by and adjusted to previous TOC demands, specifically by the previous trials’ task-order transition. Strobach and colleagues (Strobach et al., 2021b, 2023; Strobach & Wendt, 2022) demonstrated reduced order switch costs in Trial *N* when Trial *N* − 1 itself required a task-order switch (i.e., when the task order in Trial *N* − 1 changed from the penultimate Trial *N* − 2) compared with when Trial *N* − 1 required a task-order repetition (i.e., when the task order on Trial *N* − 1 was the same as in the penultimate Trial *N* – 2; Table 1). This coordination adjustment effect (i.e., CA effect) was observed across both tasks of the trial, affected RTs, error rates, and response reversal rates (i.e., rates of trials where the response order is different to the order of stimulus presentation), and occurred not only in two-choice tasks but also in three-choice tasks, for relatively short and relatively long SOAs, for relatively short and long intertrial intervals, as well as in dual tasks with and without differences in the dominance between the two individual tasks. Taken together, the CA effect represents a rather stable and replicable phenomenon. However, the mechanisms underlying CA (as indicated by the CA effect) still lack specification.

### Practice effects on dual-task coordination

When focusing on studies with investigations of practice effects on dual-task coordination, dual-task performance is usually improved after dual-task practice (with simultaneously practiced tasks under dual-task conditions) in comparison to dual-task performance after single-task practice (with tasks practiced separately under conditions of single tasks; Strobach & Schubert, 2017). This performance improvement is explained by the improvement of the coordination of two simultaneously presented and overlapping component tasks, associated with the optimization of dual-task coordination skills (Kramer et al., 1995; Maquestiaux et al., 2004). These skills are acquired during dual-task practice but not during single-task practice. Strobach (2020) summarized research on this *dual-task practice advantage hypothesis* from different dual-task practice areas that demonstrated this skill acquisition. However, according to the author’s knowledge, there is no empirical investigation on the validity of the dual-task practice advantage hypothesis for the case of TOC in the PRP context. Therefore, the first aim of the present study is to investigate whether dual-task practice, in contrast to single-task practice, leads to improved coordination of the correct dual-task order.

The investigation of dual-task practice effects could be a first empirical approach to theoretically disentangle the sets of processes of TOC and CA, as proposed by the framework of dual-task coordination adjustment (Strobach, in press). The *separability hypothesis* in this framework assumes that TOC and CA are separable sets of processes. This separability could be indicated by the different characteristics of both sets. For instance, the characteristics could differ according to the sensitivity to practice effects, so that TOC is sensitive to those effects (as predicted by the dual-task practice advantage hypothesis), while CA is not.

Findings from attentional adjustment during task-switching practice were consistent with this latter assumption of no practice effects on CA (Strobach et al., 2020). In detail, the task switching costs to switch between two sequential component tasks were reduced with practice, indicating improved task-switching performance. However, the congruency effect of task-switching stimuli (i.e., the performance difference between stimuli that map on the same response and stimuli that map on different responses) is an indicator for CA during task switching. This task switching CA was robust to the impact of practice and was not substantially improved during four practice sessions (Strobach et al., 2020). Although the practice-related improvement of task-switching performance and the lacking change of CA during task-switching practice provide preliminary evidence for different characteristics of task coordination and its adjustment, the literature does not provide pivotal tests for these assumptions in the context of dual tasks, TOC, and CA. Also, the previous literature on dual-task CA is merely at an infant stage in specifying the mechanisms of this type of dual-task meta-control (Strobach, in press). Therefore, the second aim of the present study is to further develop our understanding of these mechanisms.

## Method

### The present study

In the present study, a dual-task practice group performed arbitrary component tasks (Ruthruff et al., 2006; see also Footnote 1) in dual tasks with randomly varying task orders (i.e., random-order dual tasks; Strobach et al., 2021b). However, in contrast to merely cross-sectional investigations of order switch costs and the CA effect within one session, this group of participants practiced random-order dual tasks for four sessions. This allows analyzing the order switch costs and the CA effect longitudinally during dual-task practice. An additional group of participants also performed dual tasks with randomly varying task orders in a final session (i.e., Session 4). However, this group basically practiced the individual tasks under single-task conditions in the first three sessions (i.e., the single-task practice group). Improved TOC would be indicated by a reduction in order switch costs (1) from the beginning to the end of dual-task practice and (2) in Session 4 after dual-task practice in comparison to Session 4 after single-task practice. While there could be a reduction in order switch costs with dual-task practice/after dual-task practice versus single-task practice, according to the dual-task practice advantage hypothesis (Strobach, 2020), the CA effect does not change with practice, according to the separability hypothesis (Strobach, in press).

### Participants

Participants were students from the Medical School Hamburg and other universities in the Hamburg area, recruited via online databases and personal contacts. All participants were German native speakers and right-handed, as investigated with the Edinburgh Handedness Inventory (Oldfield, 1971); handedness is illustrated in the form of the handedness laterality quotient, where values between 1 (*very low laterality*) and 100 (*full laterality*) reflect right-handedness. They reported normal or corrected-to-normal hearing and vision and received course credit for their participation. All procedures performed in this study involving human participants were in accordance with the ethical standards of the institutional and/or national research committee and with the 1964 Helsinki Declaration and its later amendments or comparable ethical standards. The study was approved by the ethical committee of the Medical School Hamburg. Informed consent was obtained from all participants before the commencement of the study.

An a priori power analysis assuming a large within-subjects CA interaction effect size (Cohen’s *f* = 0.40; Strobach et al., 2021b), number of measures = 6, number of groups = 1, and power of 1 − ß = 0.90 suggested a sample size of *N* = 23, which I increased by one participant to create a balanced design. Therefore, I recruited 24 participants for the dual-task practice group (16 females, eight males, mean age = 22.6 years, age range: 20–28 years; mean handedness laterality quotient = 74.0, handedness laterality quotient range: 5.3–100.0). To have an equal number of participants in the single-task practice group, I included 24 participants in the single-task practice group (15 females, nine males, mean age = 21.8 years, age range: 19–29 years; mean handedness laterality quotient = 80.8, handedness laterality quotient range: 10.8–100.0).

### Apparatus

Visual stimuli in the following experiments were presented on a 22-inch color monitor (refresh rate: 60 Hz; viewed from a distance of approximately 60 cm), and auditory stimuli were presented via headphones that were connected to IBM-compatible personal computers. Experiments were controlled by the experimental software package Presentation (Version 18). Manual responses were given on QWERTZ keyboards.

### Stimuli

Participants performed a visual and an auditory choice RT task in the present dual-task situation. The auditory task included the presentation of sine-wave tones with pitches of either 350 or 1650 Hz. Participants responded with the index (*M* key) and middle (*,* key) fingers of the right hand, respectively. The visual task included the presentation of small and large visually presented triangles and responses with the middle (*X* key) and index (*C* key) fingers of the left hand, respectively.

### Procedure and design

Participants performed single-task blocks in which only one of the two tasks was presented. They also performed dual-task blocks that included the presentation of both tasks. Trials of single-task blocks started with the presentation of three dashes next to each other, of which the middle dash was located at the center of the screen. The dashes remained on the screen until the end of each trial, while they disappeared between trials. An auditory stimulus (i.e., a sine-wave tone) appeared for 100 ms in auditory single-task block trials, or a visual stimulus (i.e., a triangle) appeared centrally in the visual single-task block trials 500 ms after the onset of the presentation of the dashes and thus trial start; visual stimuli were presented until response or a maximum of 2,500 ms.

Similar to single-task trials, dual-task block trials started with the presentation of three white dashes that remained on-screen until the end of each trial (or a maximum of 4,500 ms) and disappeared between trials. After 500 ms, a first stimulus (i.e., auditory or visual) was presented, followed by the presentation of the second stimulus (i.e., visual or auditory). The constant interval between the onsets of both stimuli (i.e., SOA) was 400 ms. Incorrect single-task and dual-task trials were completed with error feedback (German word: “Fehler”) for 1,500 ms; incorrect trials included wrong or omitted responses as well as response reversals.

Single-task blocks consisted of 32 single-task trials, and stimuli were presented with equal frequency in a random order. In all 32 trials of the dual-task blocks, auditory and visual stimuli were presented with equal frequency, and stimuli were selected randomly. Participants were instructed to respond as quickly and accurately as possible in single-task blocks as well as in dual-task blocks. Additionally, in all dual-task blocks, participants were instructed that the stimuli are presented in a certain order and that task responses should be performed sequentially in an order consistent with the stimulus presentation order; by this instruction, participants gave priority to T1. In detail, they were instructed that if the auditory stimulus is presented first and the visual stimulus is presented second, then the response to the auditory task should be performed first and the response to the visual task should be performed second. In contrast, if the visual stimulus is presented first and the auditory stimulus is presented second, then the response to the visual task should be performed first and the response to the auditory task should be performed second.

**Fig. 1:**
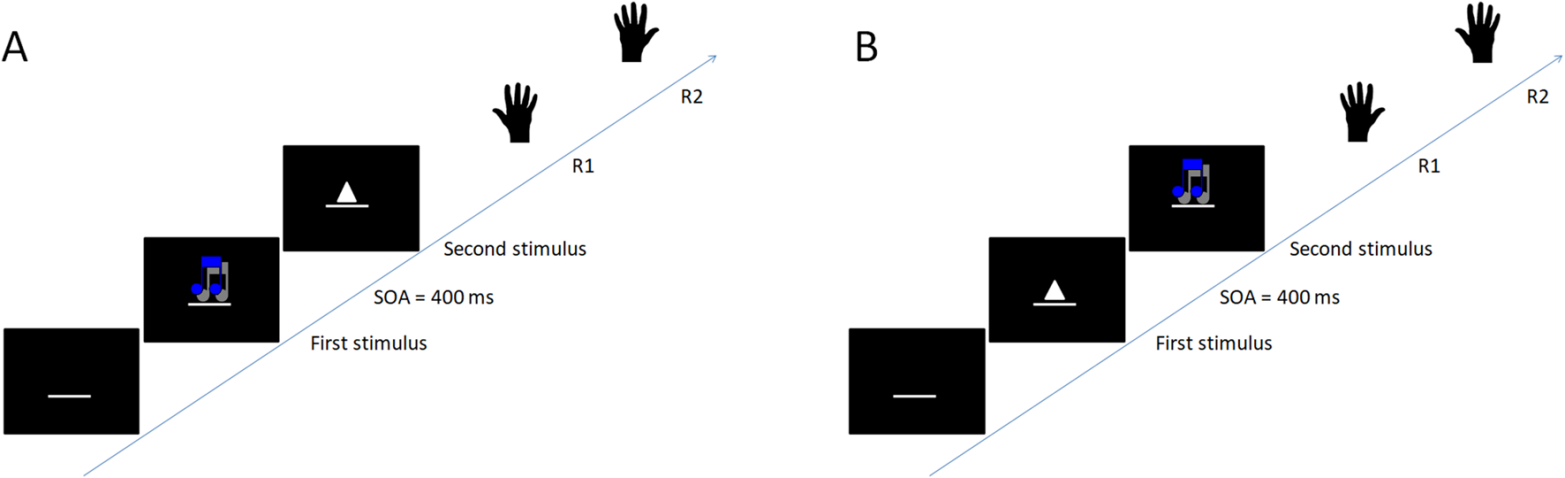
Illustration of dual-task trials. Panel (**A**): auditory-visual task order. Panel (**B**): visual-auditory task order. SOA = stimulus onset asynchrony. R1 = first response. R2 = second response

**Fig. 2:**
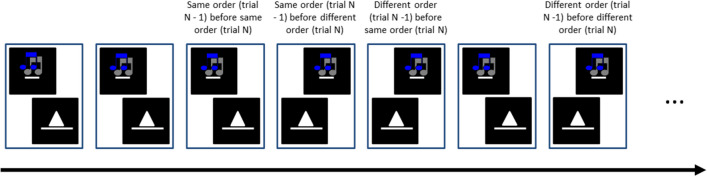
Illustration of an exemplary sequential order of dual-task trials across blocks (from left to right). In each box, the stimulus further to the left and the stimulus further to the right represent the firstly and the secondly presented task, respectively

Across the groups of this study, we conducted two different types of dual-task blocks with different task orders: dual-task blocks with random task order (*random-order dual tasks*) and dual-task blocks with fixed task order (*fixed-order dual tasks*). In random-order dual-task blocks, dual-task trials with a first auditory stimulus (auditory-visual order trials) and with a first visual stimulus (visual-auditory order trials) were randomly mixed. As a result, on some trials, the firstly presented stimulus and associated response hand repeat from trial to trial (so does the secondly presented stimulus and associated response hand), while on other trials, the firstly presented stimulus and associated response hand switch from trial to trial (so does the secondly presented stimulus and associated response hand; see Fig. 1), resulting in same-order and different-order trials (see Fig. 2). Participants had to derive the required response order merely from the stimulus presentation order; there was no cue informing them about the task order. Fixed-order dual-task blocks included trials in a constant, fixed order. This order was verbally instructed to participants before the block started. These blocks either included only trials with a first visual stimulus and a second auditory stimulus or only trials with a first auditory stimulus and a second visual stimulus.

As illustrated in Table 2, for the dual-task practice group, at the beginning of each experimental session, one visual and 1 auditory single-task block were presented. Whereas half of the participants started with a visual block, followed by an auditory block, the remaining participants performed the blocks in the opposite order. Following, two dual-task blocks with a fixed task order were conducted (fixed-order dual-task block). Whereas half of the participants started with a dual-task block and trials with a first auditory stimulus, followed by a block with trials with a first visual stimulus, the remaining participants performed the blocks in the opposite order. After this, 20 random-order dual-task blocks were performed. Participants in the dual-task practice group performed 4 experimental sessions within a 2-week time interval, with all sessions conducted on separate days. Each experimental session lasted approximately 60 minutes.

For the single-task practice group, identical to the dual-task practice group, Session 1 started with one visual and one auditory single-task block. Whereas half of the participants started with a visual block, followed by an auditory block, the remaining participants performed the blocks in the opposite order. Following, two dual-task blocks with a fixed task order were conducted (fixed-order dual-task block). Whereas half of the participants started with a dual-task block with trials with a first auditory stimulus, followed by a block with trials with a first visual stimulus, the remaining participants performed the blocks in the opposite order. These two fixed-order dual-task blocks were followed by two random-order dual-task blocks. On these two random-order dual-task blocks, the dual-task pretest in the single-task practice group (see Blocks 5 and 6 in Session 1, Table 2) was performed.^2^ (Equivalently, the pretest in the dual-task practice group was performed on their first two random-order dual-task blocks; i.e., Blocks 5 and 6 in Session 1, as illustrated in Table 2). Session 1 was completed by nine auditory and nine visual single-task blocks (each block including 64 single-task trials). Sessions 2 and 3 included 11 of those auditory and 11 of those visual single-task blocks after one visual and one auditory single-task block with only 32 trials. The modality of the single-task blocks of Sessions 1 to 3 was alternated between blocks. In total, the number of stimulus contacts during practice Sessions 1 to 3 was identical in the dual-task and single-task practice groups, so that each task was practiced equally often across the two groups (e.g., Liepelt et al., 2011; Schubert & Strobach, 2018). Session 4 of the single-task practice group was identical to Session 4 of the dual-task practice group.

### Data analyses

In both groups of participants, only trials from the random-order dual-task blocks were analyzed. Before analyzing RTs, error rates, and response reversal rates, we excluded the first two trials of each random-order dual-task block. Before the RT analysis, there was an exclusion of trials with errors in the choice decisions within the component tasks and trials with response reversals. Trials were counted as response reversals irrespective of the correctness of the choice decisions within the component tasks and irrespective of whether the response was performed before or after the presentation of the second stimulus. RTs were not trimmed. RTs, error rates, and response reversals were aggregated into four conditions (Table 1, Fig. 2): (1) a same-order trial (Trial *N* − 1) before a same-order trial (Trial *N*), (2) a different-order trial (Trial *N* − 1) before a same-order trial (Trial *N*), (3) a same-order trial (Trial *N* − 1) before a different-order trial (Trial *N*), as well as (4) a different-order trial (Trial *N* − 1) before a different-order trial (Trial *N*). Across the first and last practice sessions exclusively in the dual-task practice group, these four conditions were analyzed in frequentist methods of repeated-measures analyses of variance (ANOVAs), including the within-subjects factors CURRENT ORDER (same-order versus different-order trial in Trial *N*), PREVIOUS ORDER (same-order versus different-order trial in Trial *N* − 1), and SESSION (Session 1 versus Session 4). In a second set of analyses including not only the dual-task practice group but in both practice groups, the four conditions were analyzed in mixed-measures ANOVAs, including the within-subjects factors CURRENT ORDER (same-order versus different-order trial in Trial *N*) and PREVIOUS ORDER (same-order versus different-order trial in Trial *N* − 1) as well as the between-subjects factor GROUP (dual-task practice group versus single-task practice group). These ANOVAs were performed on T1 (the first presented task, irrespective of either auditory or visual task) and T2 (the second presented task, irrespective of either auditory or visual task): RT1 (RT of T1), RT2 (RT of T2), Error1 (errors in T1), Error2 (errors in T2), and response reversals. Note that response reversals cannot be analyzed separately for T1 and T2.

TOC is indicated by a main effect of CURRENT ORDER, while an effect of practice on TOC would be indicated by an interaction of CURRENT ORDER and SESSION (in the dual-task practice group) and an interaction of CURRENT ORDER and GROUP at the end of practice during Session 4 (in the dual-task practice group and the single-task practice group). Potential group differences in TOC at the end of practice might be explained by differences at the beginning of practice. Therefore, I compared the TOC performance in Session 1’s first two random-order dual-task blocks in the dual-task practice group and the single-task practice group (dual-task pretest) in the supplementary material. Lacking evidence for TOC differences at the beginning of practice would be indicated by a lacking main effect of GROUP and a lacking GROUP modulation of the CURRENT ORDER factor during the dual-task pretest (Table 1 in the Supplementary Material). Potential practice effects within Session 4 following dual-task and single-task practice are analyzed with frequentist methods of mixed-measures ANOVAs, including the within-subjects factors BLOCK (Blocks 1 to 10) and CURRENT ORDER (same-order versus different-order trial in Trial *N*), as well as the between-subjects factor GROUP (dual-task practice group versus single-task practice group). An interaction of these factors could be evidence for such a within-session practice effect, while the lack of this interaction does not provide evidence for this effect.

A sequential adjustment of TOC (i.e., CA) would generally be indicated by an interaction of CURRENT ORDER and PREVIOUS ORDER. A practice effect on this CA would be indicated by an additional modulation of this interaction (1) by the factor SESSION (in the dual-task practice group) and (2) by the factor GROUP at the end of practice during Session 4 (in the dual-task practice group and the single-task practice group). In cases of nonsignificant factor combinations SESSION × CURRENT ORDER, SESSION × CURRENT ORDER × PREVIOUS ORDER, GROUP × CURRENT ORDER, as well as GROUP × CURRENT ORDER × PREVIOUS ORDER (i.e., the relevant practice effects on TOC and CA), I additionally computed Bayesian factors (BF_01_) using JASP 0.18.1 with its default JASP Cauchy prior (JASP-Team, 2023). Furthermore, the Supplementary Material includes (1) separate *t* tests between same order versus different order in current trials under same-order conditions and different-order conditions in previous trials, as well as (2) separate *t* tests between same order versus different order in previous trials under same-order conditions and different-order conditions in current trials. These *t* tests were performed for RT1, RT2, Error1, Error2, as well as response reversals in Session 1 of the dual-task practice group and Session 4 of the dual-task and single-task practice groups (see section “Details of the combination of PREVIOUS TRIAL and CURRENT TRIAL separated by session and group” in the Supplementary Material).

**Fig. 3:**
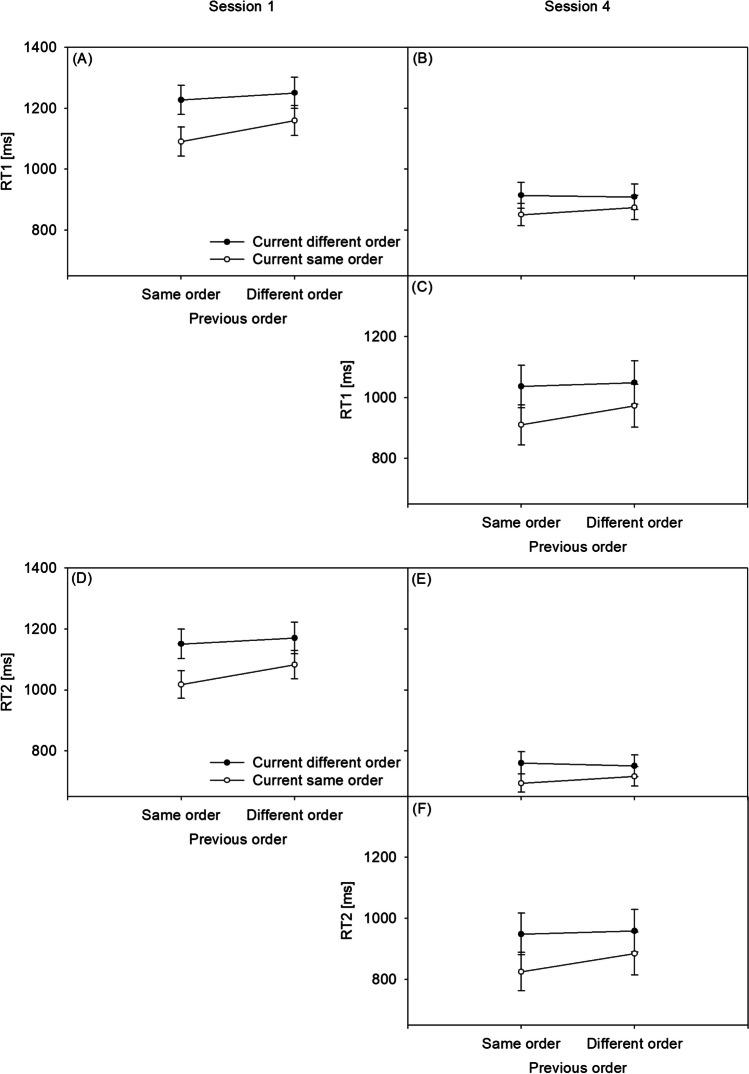
Reactions times of the first task (RT1) and of the second task (RT2) in ms. Panel (**A**): RT1 in the dual-task practice group in Session 1. Panel (**B**): RT1 in the dual-task practice group in Session 4. Panel (**C**): RT1 in the single-task practice group in Session 4. Panel (**D**): RT2 in the dual-task practice group in Session 1. Panel (**E**): RT2 in the dual-task practice group in Session 4. Panel (**F**): RT2 in the single-task practice group in Session 4

**Fig. 4:**
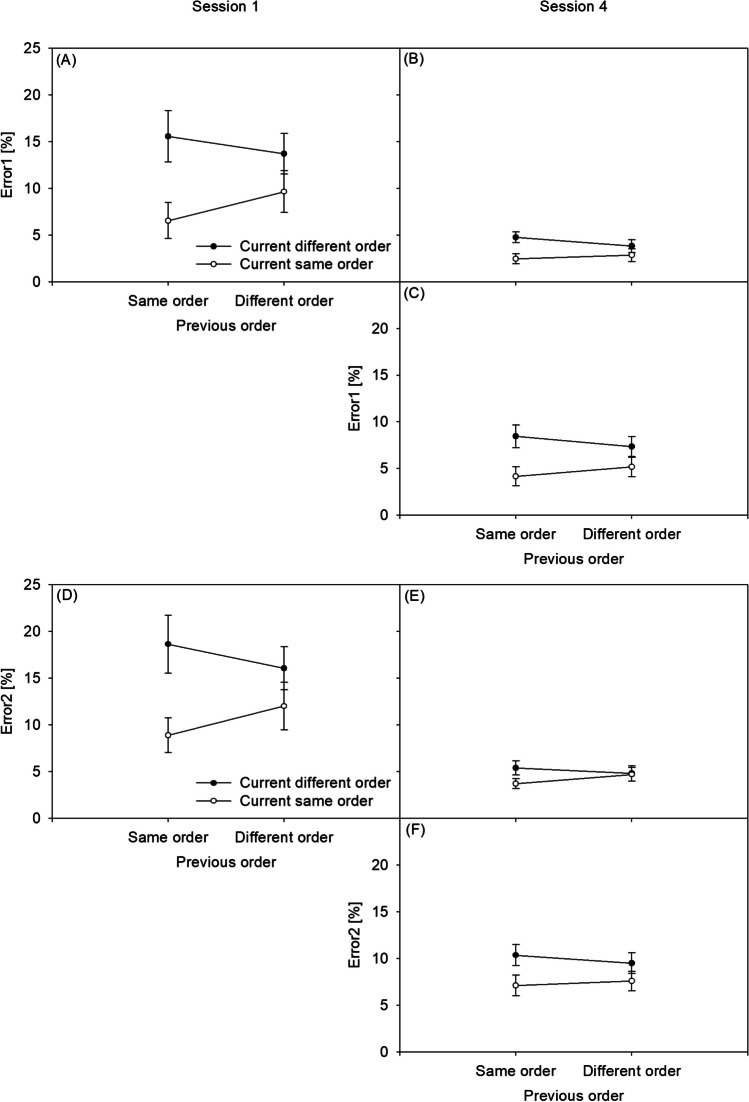
Error rates of the first task (Error1) and of the second task (Error2) in percent (%). Panel (**A**): Error1 in the dual-task practice group in Session 1. Panel (**B**): Error1 in the dual-task practice group in Session 4. Panel (**C**): Error1 in the single-task practice group in Session 4. Panel (**D**): Error2 in the dual-task practice group in Session 1. Panel (**E**): Error2 in the dual-task practice group in Session 4. Panel (**F**): Error2 in the single-task practice group in Session 4

**Fig. 5:**
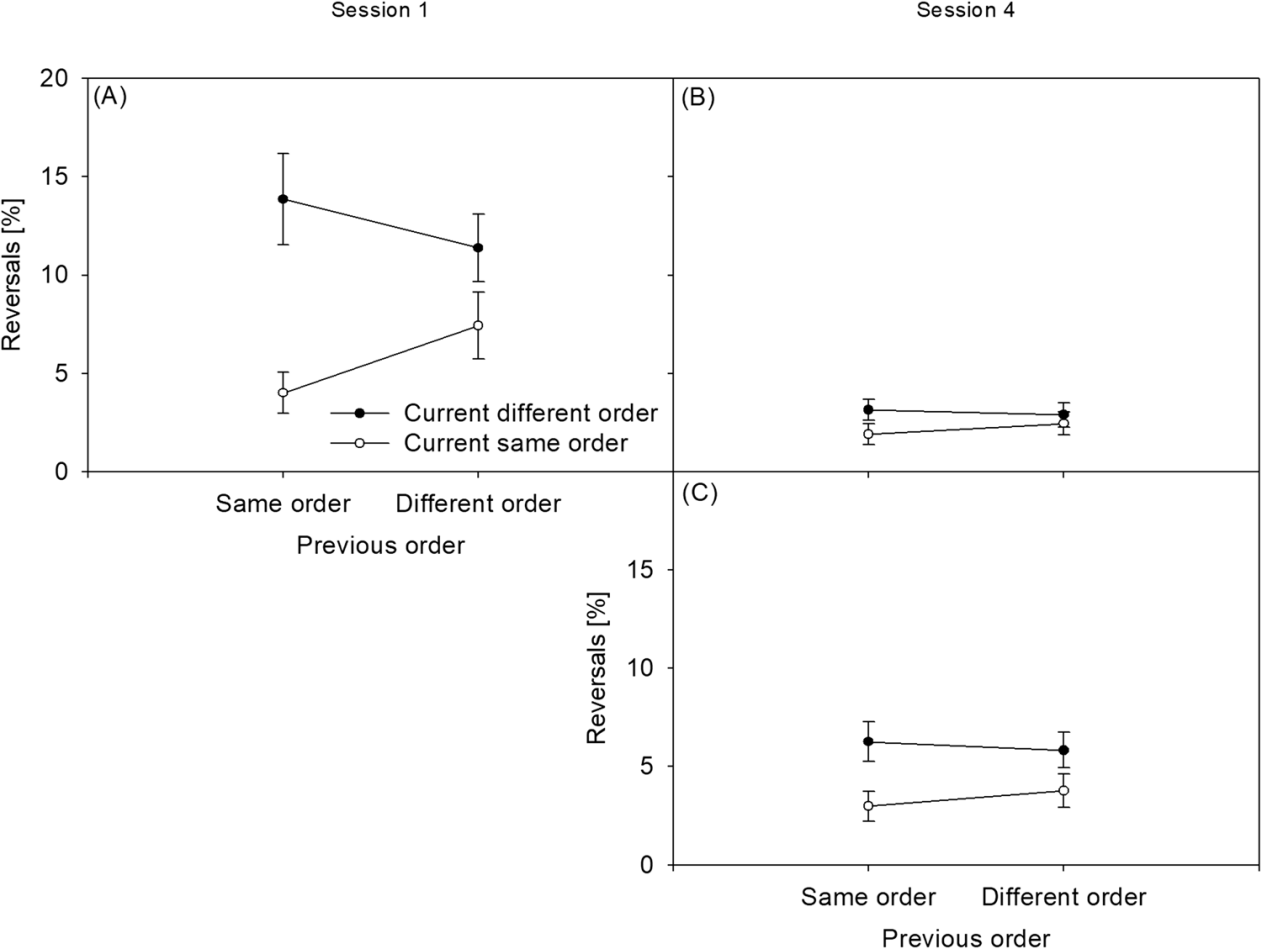
Reversal rates in %. Panel (**A**): Reversal rates in the dual-task practice group in Session 1. Panel (**B**): Reversal rates in the dual-task practice group in Session 4. Panel (**C**): Reversal rates in the single-task practice group in Session 4

RT1 of the dual-task practice group’s Sessions 1 and 4 is presented in Fig. 3A and B, respectively, while RT1 of the single-task practice group’s Session 4 is presented in Fig. 3C. RT2 of the dual-task practice group’s Sessions 1 and 4 is presented in Fig. 3D and E, respectively, while RT2 of the single-task practice group’s Session 4 is presented in Fig. 3F. Error1 of the dual-task practice group’s Sessions 1 and 4 is presented in Fig. 4A and B, respectively, while Error1 of the single-task practice group’s Session 4 is presented in Fig. 4C. Error2 of the dual-task practice group’s Sessions 1 and 4 is presented in Fig. 4D and E, respectively, while Error2 of the single-task practice group’s Session 4 is presented in Fig. 4F. Response reversal rates of the dual-task practice group’s Sessions 1 and 4 are presented in Fig. 5A and B, respectively, while response reversal rates of the single-task practice group’s Session 4 are presented in Fig. 5C. In the Supplementary Material, the order switch costs of the dual-task practice group and the single-task practice group in Sessions 1 to 4 for the RTs, error rates, and response reversal rates are illustrated in Fig. 1A, B, and C, respectively. Also in the supplementary material, the CA effects of the dual-task practice group and the single-task practice group in Sessions 1 to 4 for the RTs, error rates, and response reversal rates are illustrated in Fig. 2A, B, and C, respectively.

One participant from each group was eliminated from the final data set because of error rates during the random-order dual-task blocks of more than 30% (see also Strobach et al., 2021b; Strobach & Wendt, 2022), leaving 23 participants in each practice group. In total, the data of the dual-task practice group and single-task practice group showed 10.4% and 10.7% error trials in random-order dual-task blocks, respectively.

## Results

### RTs (Fig. 3)

Analyzing RT1 in the dual-task practice group, the main effect of CURRENT ORDER was significant, *F*(1, 22) = 52.583, *p* < .001, ŋp^2^ = .70, generally showing the present study’s order switch costs: RTs were shorter in same-order trials (*M* = 994 ms) than in different-order trials (*M* = 1,075 ms). CURRENT ORDER was modulated by SESSION, *F*(1, 22) = 14.700, *p* < .001, ŋp^2^ = .40. The reduction of order switch costs from *M* = 114 ms in Session 1 to *M* = 49 ms in Session 4 is, according to the author’s knowledge, the first demonstration of an improvement of TOC during dual-task practice.

In a between-group comparison (i.e., the dual-task versus the single-task practice group) in Session 4, CURRENT ORDER and GROUP also interacted, *F*(1, 44) = 7.871, *p* = .007, ŋp^2^ = .15. The order switch costs were reduced following dual-task practice (*M* = 49 ms) versus following single-task practice (*M* = 101 ms). Thus, experience with dual tasks in contrast with single-task experience resulted in the improvement of TOC.

Moreover, the RT1 analysis showed an interaction of CURRENT ORDER and PREVIOUS ORDER across Session 1 and 4 in the dual-task practice group, *F*(1, 22) = 9.792, *p* = .005, ŋp^2^ = .31, demonstrating a CA effect in the present study (Strobach et al., 2021b; Strobach & Wendt, 2022). However, RT1 demonstrated no evidence for a modulation of CA during practice from Session 1 to Session 4, since the combination of CURRENT ORDER, PREVIOUS ORDER, and SESSION was not significant, *F*(1, 22) < 1, BF_01_ = 2.983. Furthermore, there was an interaction of CURRENT ORDER and PREVIOUS ORDER across both groups in Session 4, *F*(1, 44) = 14.044, *p* < .001, ŋp^2^ = .24, demonstrating the CA effect. However, there is anecdotal evidence that this adjustment did not differ between the two practice groups, which is indicated by the lack of interaction between CURRENT ORDER, PREVIOUS ORDER, and GROUP in Session 4, *F*(1, 44) < 1, BF_01_ = 2.929.

Analyzing RT2 similarly to RT1 in the dual-task practice group, the main effect of CURRENT ORDER was significant, *F*(1, 22) = 48.585, *p* < .001, ŋp^2^ = .69. RTs were shorter in same-order trials (*M* = 878 ms) than in different-order trials (*M* = 958 ms). CURRENT ORDER interacted with SESSION, *F*(1, 22) = 12.725, *p* = .002, ŋp^2^ = .37. In the dual-task practice group, the reduction of order switch costs from *M* = 110 ms in Session 1 to *M* = 51 ms in Session 4 demonstrated a practice-related improvement of TOC. In a between-group comparison (i.e., the dual-task versus the single-task practice group) in Session 4, CURRENT ORDER and GROUP also interacted, *F*(1, 44) = 8.039, *p* = .007, ŋp^2^ = .15. The order switch costs were reduced following dual-task practice (*M* = 51 ms) versus following single-task practice (*M* = 99 ms), indicating improved TOC.

The RT2 analysis showed an interaction of CURRENT ORDER and PREVIOUS ORDER across sessions in the dual-task practice group, *F*(1, 22) = 9.238, *p* = .006, ŋp^2^ = .30, demonstrating the CA effect. However, RT2 demonstrated no evidence for a practice-related modulation of CA from Session 1 to Session 4, since the combination of CURRENT ORDER, PREVIOUS ORDER, and SESSION was not significant, *F*(1, 22) < 1 BF_01_ = 2.989. Furthermore, there was an interaction of CURRENT ORDER and PREVIOUS ORDER across both groups (i.e., the dual-task and the single-task practice groups) in Session 4, *F*(1, 44) = 14.818, *p* < .001, ŋp^2^ = .25. However, this CA effect did not differ between the groups, which is indicated by an at least anecdotally lacking interaction of CURRENT ORDER, PREVIOUS ORDER, and GROUP in Session 4, *F*(1, 44) < 1, BF_01_ = 2.978.

### Errors (Fig. 4)

Analyzing Error1 in the dual-task practice group, the main effect of CURRENT ORDER was significant, *F*(1, 22) = 81.444, *p* < .001, ŋ*p*^2^ = .79, generally showing order switch costs in the present study. As illustrated across Fig. 4A and B, error rates were lower in same-order trials (*M* = 5.4%) than in different-order trials (*M* = 9.2%); that is, lines with unfilled circles were basically below lines with filled circles. SESSION modulated CURRENT ORDER, *F*(1, 22) = 54.470, *p* < .001, ŋp^2^ = .71. This reduction of order switch costs from *M* = 6.5% in Session 1 to *M* = 1.1% in Session 4 is illustrated by a smaller distance between the unfilled and filled lines in Fig. 4A compared with Fig. 4B, demonstrating the practice-related improvement of TOC in the dual-task practice group.

The between-group comparison in Session 4 allows for similar conclusions. That is, CURRENT ORDER and GROUP interacted in this session, *F*(1, 44) = 7.381, *p* = .009, ŋp^2^ = .14. The order switch costs were reduced following dual-task practice (*M* = 1.1%) versus following single-task practice (*M* = 3.2%), illustrated by a smaller distance between the unfilled and filled lines in Fig. 4B compared with Fig. 4C.

Importantly, the Error1 analysis showed an interaction of CURRENT ORDER and PREVIOUS ORDER across Sessions 1 and 4 of the dual-task practice group, *F*(1, 22) = 7.423, *p* = .012, ŋp^2^ = .25, demonstrating the sequential adjustment of TOC. However, different from previous analyses, Error1 demonstrated evidence for a practice-related modulation of this CA from Session 1 to Session 4, since the combination of CURRENT ORDER, PREVIOUS ORDER, and SESSION was significant, *F*(1, 22) = 7.188, *p* = .014, ŋp^2^ = .24. While the interaction of CURRENT ORDER and PREVIOUS ORDER was significant in Session 1, *F*(1, 22) = 8.320, *p* = .009, ŋp^2^ = .27, this factor combination was not significant in Session 4, *F*(1, 22) < 1. Furthermore, across the dual-task and the single-task practice groups, there was an interaction of CURRENT ORDER and PREVIOUS ORDER in Session 4, *F*(1, 44) = 5.420, *p* = .025, ŋp^2^ = .11, demonstrating the CA effect. However, this adjustment did not differ between the two practice groups in Session 4, *F*(1, 44) = 2.847, *p* = .103, BF_01_ = 4.493.

Analyzing Error2 similar to Error1, the main effect of CURRENT ORDER was significant in the dual-task practice group, *F*(1, 22) = 56.484, *p* < .001, ŋp^2^ = .72. Error rates were lower in same-order trials (*M* = 7.3%) than in different-order trials (*M* = 11.2%); that is, lines with unfilled circles were basically below lines with filled circles in Fig. 4D and E. CURRENT ORDER was modulated by SESSION, *F*(1, 22) = 70.710, *p* < .001, ŋp^2^ = .76. Order switch costs were reduced from *M* = 6.9% in Session 1 to *M* = 0.1% in Session 4, illustrated by a smaller distance between the unfilled and filled lines in Fig. 4D compared with Fig. 4E. This finding demonstrates the practice-related improvement of TOC in the dual-task practice group.

In a between-group comparison in Session 4, CURRENT ORDER and GROUP also interacted, *F*(1, 44) = 5.325, *p* = .026, ŋp^2^ = .11. The order switch costs were reduced following dual-task practice (*M* = 0.1%) versus following single-task practice (*M* = 2.6%), illustrated by a smaller distance between the unfilled and filled lines in Fig. 4E compared with Fig. 4F. This finding demonstrates the improvement of TOC.

Similar to all previous analyses, the Error2 analysis showed an interaction of CURRENT ORDER and PREVIOUS ORDER across sessions of the dual-task practice group, *F*(1, 22) = 9.362, *p* = .006, ŋp^2^ = .30, demonstrating the sequential adjustment of task-order coordination (i.e., CA). However, the Error2 rates demonstrated no evidence for a practice-related modulation of CA from Session 1 to Session 4, since the combination of CURRENT ORDER, PREVIOUS ORDER, and SESSION did not reach significance, *F*(1, 22) = 3.563, *p* = .072, BF_01_ = 1.558. Furthermore, there was an interaction of CURRENT ORDER and PREVIOUS ORDER across the dual-task and the single-task practice groups in Session 4, *F*(1, 44) = 5.360, *p* = .025, ŋp^2^ = .11, demonstrating the CA effect. However, this adjustment did not differ between the two practice groups in Session 4, *F*(1, 44) < 1, BF_01_ = 13.197.

### Response reversals (Fig. 5)

The analysis of the response reversals revealed a main effect of CURRENT ORDER, *F*(1, 22) = 66.809, *p* < .001, ŋp^2^ = .75, showing lower response reversal rates in same-order (*M* = 3.9%) than in different-order trials (*M* = 7.8%) in the dual-task practice group. The factor SESSION modulated CURRENT ORDER, *F*(1, 22) = 56.771, *p* < .001, ŋp^2^ = .72. The reduction of order switch costs from *M* = 6.9% in Session 1 to *M* = 0.1% in Session 4 indicated the improvement of TOC with dual-task practice. In a between-group comparison in Session 4, CURRENT ORDER and GROUP interacted, *F*(1, 44) = 6.585, *p* = .014, ŋp^2^ = .13. The order switch costs were reduced following dual-task practice (*M* = 0.1%) versus following single-task practice (*M* = 2.7%), explained by the improvement of TOC.

Similar to all previous analyses, the reversal rate analysis showed an interaction of CURRENT ORDER and PREVIOUS ORDER across sessions in the dual-task practice group, *F*(1, 22) = 10.140, *p* = .004, ŋp^2^ = .32, demonstrating the impressive robustness of the finding of a sequential adjustment of TOC. However, the reversal rates demonstrated no evidence for a practice-related modulation of this CA during dual-task practice from Session 1 to Session 4, since the combination of CURRENT ORDER, PREVIOUS ORDER, and SESSION was not significant, *F*(1, 22) = 1.131, *p* = .253, BF_01_ = 3.276. Furthermore, there was a trend for an interaction of CURRENT ORDER and PREVIOUS ORDER across both groups in Session 4, *F*(1, 44) = 3.717, *p* = .060, ŋp^2^ = .08, demonstrating a weak trend CA. However, this trending adjustment did not differ between the two practice groups, which is indicated by a lacking interaction of CURRENT ORDER, PREVIOUS ORDER, and GROUP in Session 4, *F*(1, 44) < 1, BF_01_ = 9.959.

## Discussion

The aim of the present study was to investigate the characteristics of TOC in dual-task situations. In particular, this study was interested in the effects of practice on TOC and on the sequential adjustment (i.e., CA) of TOC. The results demonstrated the existence of processes of TOC (Kübler et al., 2022a, 2022b; Szameitat et al., 2006) and CA (Strobach et al., 2021b, 2023; Strobach & Wendt, 2022) in a practice context. However, practice mostly had different effects on these sets of processes; these differences are outlined below.

## Discussing practice effects on task-order coordination

Order switch costs were reduced, and TOC improved (1) during dual-task practice and (2) in contrast to the effects of single-task practice, based on the effects with frequentist methods of repeated-measures and mixed-measures ANOVAs, respectively. The TOC improvement in contrast to single-task practice shows that experience in the individual component tasks and related shortening of the task processing stages (Ruthruff et al., 2006; Strobach et al., 2013) cannot explain this TOC improvement completely. In this case, the practice-related shortening of the processing stages could be somewhat equivalent to a practice-related prolonging of SOAs in relation to the durations of the task processing stages (which would make the identification of the stimulus presentation order easier). However, if this were the case, the practice effects on TOC would be similar after dual-task and single-task practice (Ruthruff et al., 2006). In contrast, the finding of an improvement in TOC is consistent with the dual-task practice advantage hypothesis (Strobach, 2020) and extends this hypothesis to the component of dual-task order coordination: TOC is improved because dual-task practice induces participants to adopt a strategy of coordinating the two tasks, which enables them to develop dual-task skills of cognitive resource allocation and scheduling.

The general evidence of TOC in the present study also helps to specify the characteristics of the combined component tasks. TOC is exclusively relevant in dual tasks combining component tasks that include bottleneck processes, while TOC would not be relevant in bottleneck-free component tasks. While there is first evidence for the lack of bottleneck processing of ideomotor tasks at low levels of practice (Maquestiaux et al., 2020), the evidence for TOC in the present PRP-like dual-task situation points to component tasks that include such processing. Thus, the present component tasks might not be ideomotor-compatible tasks but rather arbitrary tasks.

The present data are also informative about the mechanisms underlying TOC and where the TOC processes are located within dual-task processing. In the present dual tasks combining component tasks that include bottleneck processes, if TOC processes are located before the bottleneck stage of T1, then the effects of TOC in T1 would propagate to T2 and TOC should have similar effects on T1 and T2. To test this prediction, in the dual-task practice group across Session 1 and 4, there should be similar effects of CURRENT ORDER and CURRENT ORDER × SESSION on RT1 and RT2. In a combined analysis of the dual-task practice group and the single-task practice in Session 4, the effects should be similar for CURRENT ORDER and CURRENT ORDER × GROUP. The first analysis within the dual-task practice group was consistent with this prediction and showed similar effects on RT1 and RT2, *F*s(1, 22) < 1.129, *p*s < .300. Similarly, the second analysis in the dual-task practice group and the single-task practice group was also consistent with this prediction and showed similar effects on RT1 and RT2, *F*s(1, 22) < 1. This set of data points to the location of TOC processes before the bottleneck stage of T1.

## Discussing practice effects on task-order coordination adjustment

In contrast to the findings of practice effects on TOC, practice across four sessions did not show substantial evidence for an effect on the CA effect and thus CA in the RT data and the response reversals. This lacking evidence is at least based on weak conclusions of the applied Bayes methods. Exclusively, the analysis of Error1 data showed that the CA effect was modulated by practice (this modulation cannot completely be excluded for the Error2 data). This modulation is surprising since error data are usually less sensitive to modulations in dual-task performance compared with RTs (Strobach et al., 2015). The modulation of CA with practice might result from the disappearance of the interaction of PREVIOUS ORDER and CURRENT ORDER from Sessions 1 to 4. Probably, the disappearing interaction in Session 4 results from a floor effect. The Error1 data in Session 4 of the dual-task practice groups revealed very low error values, which might have prevented the illustration of this interaction in this practice group. This could also explain why the interaction of CURRENT ORDER and PREVIOUS ORDER was only marginally significant in the analysis of the response reversal rates during dual-task practice. In particular, the reversal rates in the dual-task practice group were relatively low. Despite these latter phenomena and the less clear results concerning practice effects on CA, the overall argument sustains that TOC and CA show differentiable result patterns with practice, indicative of separable sets of mechanisms (i.e., the separability hypothesis). However, in future studies, there is a need to further confirm whether TOC and CA can be dissociated (given that the present evidence rests on a single-experiment study and is less strong for the error rates) based on differential training effects.

The present data might also be informative about the mechanisms underlying CA and the location of CA-related processes within dual-task processing. In the present dual tasks combining component tasks that include bottleneck processes, if CA processes are located before the bottleneck stage of T1, then the effects of CA in T1 would propagate to T2 and CA should have similar effects on T1 and T2. To test this prediction, in the dual-task practice group across Session 1 and 4, there should be similar effects of CURRENT ORDER × PREVIOUS ORDER and CURRENT ORDER × PREVIOUS ORDER × SESSION on RT1 and RT2. In a combined analysis of the dual-task practice group and the single-task practice in Session 4, the effects should be similar for CURRENT ORDER x PREVIOUS ORDER and CURRENT ORDER × PREVIOUS ORDER × GROUP. The first analysis within the dual-task practice group was consistent with this prediction and showed similar effects on RT1 and RT2, *F*s(1, 22) < 1. Similarly, the second analysis in the dual-task practice group and the single-task practice group was also consistent with this prediction and showed similar effects on RT1 and RT2, *F*s(1, 22) < 1. This set of data points to the location of CA processes before the bottleneck stage of T1.

In general, several models of dual-task performance discuss task coordination processes. For example, the executive-process interactive control (EPIC) architecture (Meyer & Kieras, 1997) assumes dual-task performance involves coordination of processes (e.g., temporarily locking out processing for one task to avoid conflict in processing for another task). The executive control theory of visual attention (ECTVA; Logan & Gordon, 2001) assumes the strategic setting of component-task parameters to enable dual-task performance that minimizes crosstalk. Adaptive control of thought—rational (ACT-R) of dual-task performance (e.g., Anderson et al., 2005; Byrne & Anderson, 2001) characterizes how component-task processes can be organized within an integrated cognitive architecture. Subsequent developments such as threaded cognition (e.g., Salvucci & Taatgen, 2008) built on that earlier work to show how dual tasks can be coordinated as distinct "threads" of information processing. Although these models make very detailed mechanistic and/or computational assumptions about dual-task performance and active task coordination, they rarely point to a cognitive metacontrol level in addition to the active dual-task coordination processes (Strobach, in press). One of the rare exceptions is the EPIC model, which proposes the daring and cautious adjustment of dual-task coordination (Meyer & Kieras, 1997).

However, what exactly is CA, irrespective of different practice levels? And how is this exact explanation indicated by the data? One explanation for CA and the CA effect might refer to task-order transition sets (Steinhauser et al., 2021). A transition set is a cognitive representation of abstract control states. These control states are assumed to reflect control parameters needed to meet the processing demands and participants’ expectations in a current trial—namely, to switch or repeat a task order. They are abstract because they are independent of the specific task orders they operate on (Dignath & Kiesel, 2021). More specifically, there might be a task-order switch transition set including the control parameters for a task-order switch and a task-order repetition transition set encompassing the control parameters for a task-order repetition. Performance should be impaired in trials in which participants have to implement a new task-order transition set in working memory compared with trials in which participants can apply the previous task-order transition set again. Accordingly, any repetition of the task-order transition set should result in a performance benefit. Looking at the generic forms of the interaction of the factors CURRENT RIAL and PREVIOUS TRIAL in each group and session, these forms are basically consistent with this assumption. This is because in the analyses of these forms as reported in the supplementary material, at least responses in repeated task orders in Trial *N* were significantly improved after order repetitions than after an order switch in trial *N* − 1 (responses in different task orders in Trial *N* after an order switch and after an order repetition basically did not differ). For instance, there is the observation that people are faster when task order is AAA (task-order repetition after task-order repetition) than when task order is BBA (task-order switch after task-order repetition).

From a general perspective, the lack of practice effects on CA is different from the general finding of effects of practice, experience, and training in other domains of cognitive control and beyond (for an overview, see Strobach & Karbach, 2021). The current situation might be one of the few situations in which practice was not effective in changing task performance. This conclusion is similar to the lack of practice effects on attentional adjustment (Strobach et al., 2020), making practice an informative tool to investigate the characteristics of specific cognitive mechanisms, processes, and domains.

## Summary

In sum, the present study demonstrated the existence of TOC and CA in dual tasks. It further showed practice effects on TOC and no substantial practice effects on CA. These different practice characteristics are consistent with the assumptions of the separability hypothesis. This hypothesis assumes that TOC and CA represent separable sets of mechanisms (Strobach, in press).

**Table 1:** Overview of the different types of trial sequences with the example of the task order AB in the current trial

Previous trial N - 2	Previous trial N - 1	Current trial N	Task-order sequence
AB	AB	AB	Condition 1: same order before same order
BA	AB	AB	Condition 2: different order before same order
BA	BA	AB	Condition 3: same order before different order
AB	BA	AB	Condition 4: different order before different order

AB and BA represent two different types of task orders in dual-task trials. Reduced order switch costs between Conditions 4 versus 2 in relation to the cost differences between Conditions 3 versus 1 show the coordination adjustment (CA) effect

**Table 2:** Overview of the block order in Session 1 to 4 in the dual-task (DT) practice group and single-task (ST) practice group

	Session 1		Session 2		Session 3		Session 4	
Block number	DT practice group	ST practice group	DT practice group	ST practice group	DT practice group	ST practice group	DT practice group	ST practice group
1	ST block	ST block	ST block	ST block	ST block	ST block	ST block	ST block
2	ST block	ST block	ST block	ST block	ST block	ST block	ST block	ST block
3	Fixed DT block	Fixed DT block	Fixed DT block	ST block	Fixed DT block	ST block	Fixed DT block	Fixed DT block
4	Fixed DT block	Fixed DT block	Fixed DT block	ST block	Fixed DT block	ST block	Fixed DT block	Fixed DT block
5	Random DT block	Random DT block	Random DT block	ST block	Random DT block	ST block	Random DT block	Random DT block
6	Random DT block	Random DT block	Random DT block	ST block	Random DT block	ST block	Random DT block	Random DT block
7	Random DT block	ST block	Random DT block	ST block	Random DT block	ST block	Random DT block	Random DT block
8	Random DT block	ST block	Random DT block	ST block	Random DT block	ST block	Random DT block	Random DT block
9	Random DT block	ST block	Random DT block	ST block	Random DT block	ST block	Random DT block	Random DT block
10	Random DT block	ST block	Random DT block	ST block	Random DT block	ST block	Random DT block	Random DT block
11	Random DT block	ST block	Random DT block	ST block	Random DT block	ST block	Random DT block	Random DT block
12	Random DT block	ST block	Random DT block	ST block	Random DT block	ST block	Random DT block	Random DT block
13	Random DT block	ST block	Random DT block	ST block	Random DT block	ST block	Random DT block	Random DT block
14	Random DT block	ST block	Random DT block	ST block	Random DT block	ST block	Random DT block	Random DT block
15	Random DT block	ST block	Random DT block	ST block	Random DT block	ST block	Random DT block	Random DT block
16	Random DT block	ST block	Random DT block	ST block	Random DT block	ST block	Random DT block	Random DT block
17	Random DT block	ST block	Random DT block	ST block	Random DT block	ST block	Random DT block	Random DT block
18	Random DT block	ST block	Random DT block	ST block	Random DT block	ST block	Random DT block	Random DT block
19	Random DT block	ST block	Random DT block	ST block	Random DT block	ST block	Random DT block	Random DT block
20	Random DT block	ST block	Random DT block	ST block	Random DT block	ST block	Random DT block	Random DT block
21	Random DT block	ST block	Random DT block	ST block	Random DT block	ST block	Random DT block	Random DT block
22	Random DT block	ST block	Random DT block	ST block	Random DT block	ST block	Random DT block	Random DT block
23	Random DT block	ST block	Random DT block	ST block	Random DT block	ST block	Random DT block	Random DT block
24	Random DT block	ST block	Random DT block	ST block	Random DT block	ST block	Random DT block	Random DT block

The single-task blocks 1 and 2 in all sessions and groups combined only 32 trials, while the single-task blocks 7 to 24 in Session 1 and single-task blocks 3 to 22 in Session 2 and 3 in the single-task group combined 64 trials. The pre-test across both practice groups was performed on the data from Blocks 5 and 6 in Session 1. The Session 1 test in the dual-task practice group was performed on all random-order dual-task blocks in this session, while the Session 4 test in the dual-task and single-task practice groups was performed on all random-order dual-task blocks in this session. DT = dual-task. ST = single-task

## Supplementary information

Below is the link to the electronic supplementary material.

Supplementary file1 (DOC 1548 KB)

## Data Availability

Data or materials for the experiments are available (https://osf.io/gmurx/), and none of the experiments was preregistered.

## Footnotes

1 Ruthruff et al. (2006) “are aware of no previous studies with clear evidence of the elimination of the bottleneck with either compatible, arbitrary, or incompatible tasks” (p. 141). Thus, PRP paradigms with combinations of those tasks “where the stimulus codes and response codes do not resemble each other but have some natural relationship” (p. 141, compatible tasks), “where there is no pre-learned association between stimuli and responses” (p. 141, arbitrary tasks), as well as “where the mapping opposes some pre-learned association” (p. 141, incompatible tasks) should basically show PRP effects at low levels of practice. However, recent findings on combinations of ideomotor-compatible tasks “where the stimulus codes closely resemble the response codes“ (pp. 140–141) represent a specific case in which no bottleneck might be required and no PRP effect should occur (Maquestiaux et al., 2020). However, the latter type of component tasks does not require any type of task-order coordination because there is no requirement to bring bottleneck processes into a specific order. Therefore, any sign of task-order coordination is evidence against the involvement of ideomotor tasks in a PRP situation.

2 This choice for two random-order dual-task blocks for the pretest was based on the motivation to have a sufficient number of trials to assess order switch costs at the beginning of single-task practice and compare these costs with the performance at the beginning of dual-task practice. However, two random-order dual-task blocks do not provide a robust data base to assess the CA effect. To assess this effect, there would be a requirement for an increased number of blocks. However, this increase would not enable a single-task practice group with no or at least a low amount of dual-task experience before testing dual-task performance at the end of practice.

## Code availability

Experimental code for the experiments is available upon request.
