# Supplementary material for: Practice effects on dual-task order coordination and its sequential adjustment
Source: Psychon Bull Rev. 2024 Feb 26;31(5):2189–204. doi: 10.3758/s13423-024-02476-6 (PMC11543754; doi:10.3758/s13423-024-02476-6)
Supplement: Supplementary file 1 — Supplementary file1 (DOC 1548 KB) [file 13423_2024_2476_MOESM1_ESM.doc]

**Supplementary material of**

**“Practice effects on dual-task order coordination and its sequential adjustment”**

**Figure 1. Order switch costs in Session 1 to 4, in the dual-task and single-task practice groups, as well as after previous same order and different order in trial N – 1. Panel (A): Reaction times (RTs) in Task 1 and Task 2. Panel (B): Error rates in Task 1 and Task 2. Panel (C): Response reversal rates.**

**
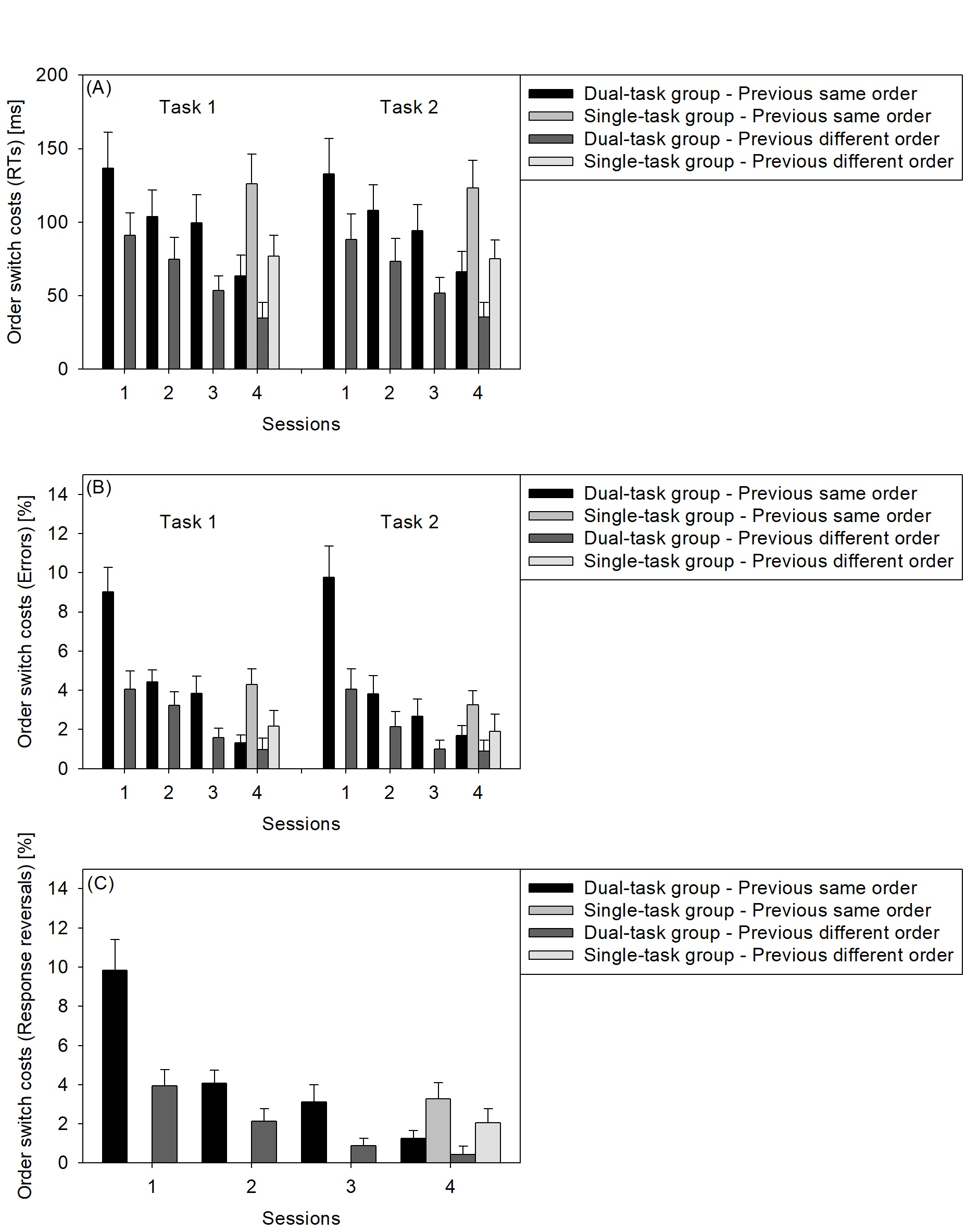
**

**Figure 2. Order switch adjustment effects in Session 1 to 4, as well as in the dual-task and single-task practice groups. Panel (A): Reaction times (RTs) in Task 1 and Task 2. Panel (B): Error rates in Task 1 and Task 2. Panel (C): Response reversal rates.**

**
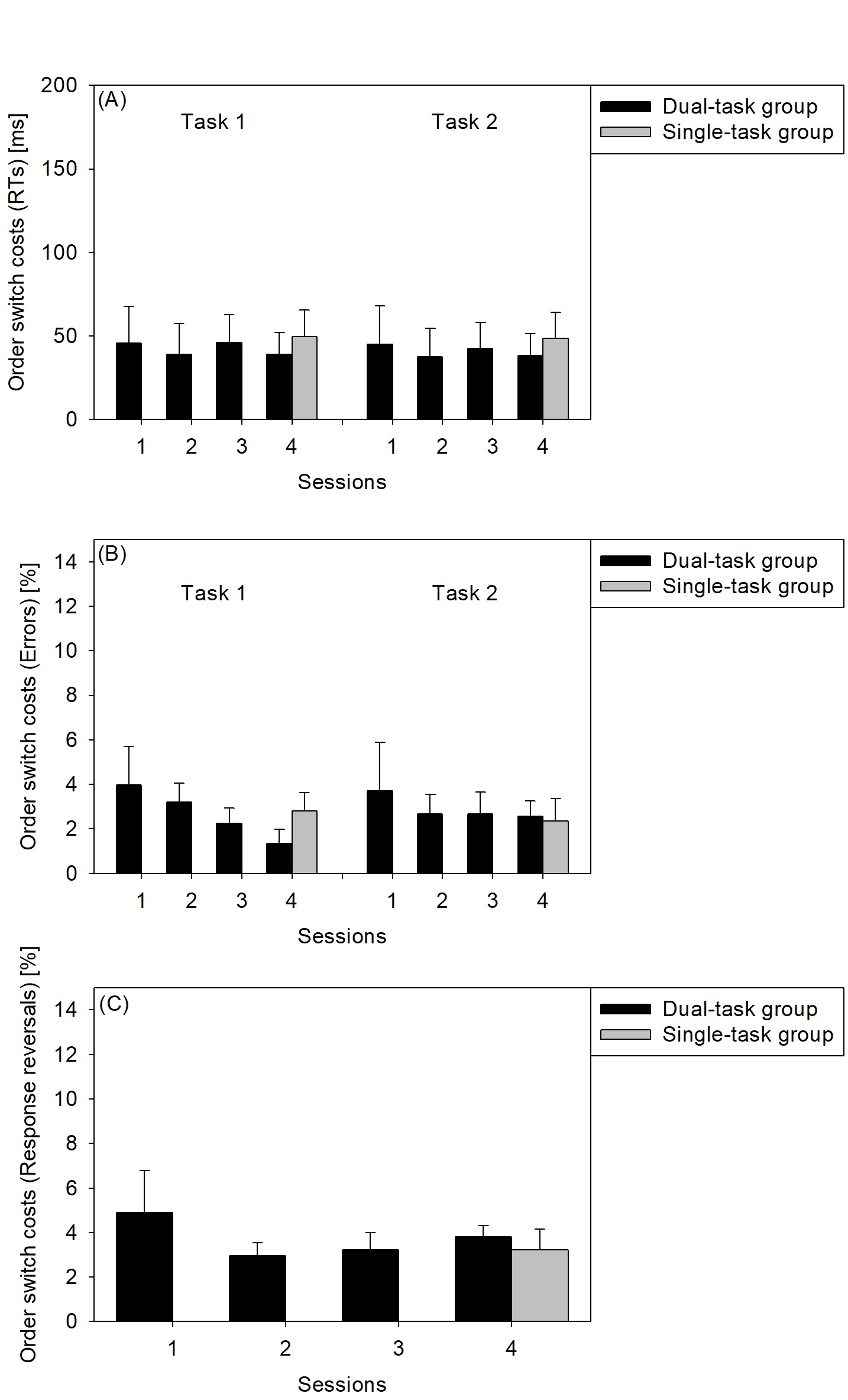
**

**Detailed results of the combination of PREVIOUS TRIAL and CURRENT TRIAL separated by session and group**

**RT1**

In Session 1 of the dual-task practice group (Figure 1A), the first set compared same-order and different-order trials in the current trials after different order trials in trial N – 1, *t*(22) = 5.627, *p* < .001, as well as after same-order trials in trial N – 1, *t*(22) = 6.152, *p* < .001. Although both tests were significant, order switch costs in trial N were substantially smaller after an order switch in trial N – 1 (*M* = 91 ms) than after an order repetition in trial N – 1 (*M* = 137 ms). The second set of tests analyzed the specific nature of this reduction in order switch costs. Responses in order repetitions in trial N were significantly slower after a different order (vs. same order) in trial N -1, *t*(22) = 5.382, *p* < .001. At the same time, RTs of different orders in trial N did not differ after a different order (vs. same order) in trial N – 1, *t*(22) = 1.473, *p* = .155.

In Session 4 of the dual-task practice group (Figure 1B), the first set compared same-order and different-order trials in the current trials after different order trials in trial N – 1, *t*(22) = 3.229, *p* < .001, as well as after same-order trials in trial N – 1, *t*(22) = 4.576, *p* < .001. Although both tests were significant, order switch costs in trial N were substantially smaller after an order switch in trial N – 1 (*M* = 35 ms) than after an order repetition in trial N – 1 (*M* = 64 ms). The second set of tests analyzed the specific nature of this reduction in order switch costs. Responses in order repetitions in trial N were significantly slower after a different order (vs. same order) in trial N -1, *t*(22) = 2.851, *p* = .009. At the same time, RTs of different orders in trial N did not differ after a different order (vs. same order) in trial N – 1, *t*(22) < 1.

In Session 4 of the single-task practice group (Figure 1C), the first set compared same-order and different-order trials in the current trials after different order trials in trial N – 1, *t*(22) = 5.357, *p* < .001, as well as after same-order trials in trial N – 1, *t*(22) = 6.303, *p* < .001. Although both tests were significant, order switch costs in trial N were substantially smaller after an order switch in trial N – 1 (*M* = 77 ms) than after an order repetition in trial N – 1 (*M* = 126 ms). The second set of tests analyzed the specific nature of this reduction in order switch costs. Responses in order repetitions in trial N were significantly slower after a different order (vs. same order) in trial N -1, *t*(22) = 4.263, *p* < .001. At the same time, RTs of different orders in trial N did not differ after a different order (vs. same order) in trial N – 1, *t*(22) = 1.400, *p* = .175.

**RT2**

In Session 1 of the dual-task practice group (Figure 1D), the first set compared same-order and different-order trials in the current trials after different order trials in trial N – 1, *t*(22) = 5.036, *p* < .001, as well as after same-order trials in trial N – 1, *t*(22) = 5.543, *p* < .001. Although both tests were significant, order switch costs in trial N were substantially smaller after an order switch in trial N – 1 (*M* = 88 ms) than after an order repetition in trial N – 1 (*M* = 133 ms). The second set of tests analyzed the specific nature of this reduction in order switch costs. Responses in order repetitions in trial N were significantly slower after a different order (vs. same order) in trial N -1, *t*(22) = 4.820, *p* < .001. At the same time, RTs of different orders in trial N did not differ after a different order (vs. same order) in trial N – 1, *t*(22) = 1.292, *p* = .210.

In Session 4 of the dual-task practice group (Figure 1E), the first set compared same-order and different-order trials in the current trials after different order trials in trial N – 1, *t*(22) = 3.409, *p* = .002, as well as after same-order trials in trial N – 1, *t*(22) = 4.842, *p* < .001. Although both tests were significant, order switch costs in trial N were substantially smaller after an order switch in trial N – 1 (*M* = 35 ms) than after an order repetition in trial N – 1 (*M* = 66 ms). The second set of tests analyzed the specific nature of this reduction in order switch costs. Responses in order repetitions in trial N were significantly slower after a different order (vs. same order) in trial N -1, *t*(22) = 2.499, *p* = .020. At the same time, RTs of different orders in trial N did not differ after a different order (vs. same order) in trial N – 1, *t*(22) < 1.

In Session 4 of the single-task practice group (Figure 1F), the first set compared same-order and different-order trials in the current trials after different order trials in trial N – 1, *t*(22) = 5.864, *p* < .001, as well as after same-order trials in trial N – 1, *t*(22) = 6.657, *p* < .001. Although both tests were significant, order switch costs in trial N were substantially smaller after an order switch in trial N – 1 (*M* = 75 ms) than after an order repetition in trial N – 1 (*M* = 123 ms). The second set of tests analyzed the specific nature of this reduction in order switch costs. Responses in order repetitions in trial N were significantly slower after a different order (vs. same order) in trial N -1, *t*(22) = 3.919, *p* < .001. At the same time, RTs of different orders in trial N did not differ after a different order (vs. same order) in trial N – 1, *t*(22) = 1.193, *p* = .267.

**Error1**

In Session 1 of the dual-task practice group (Figure 2A), the first set compared same-order and different-order trials in the current trials after different order trials in trial N – 1, *t*(22) = 4.350, *p* < .001, as well as after same-order trials in trial N – 1, *t*(22) = 7.162, *p* < .001. Although both tests were significant, order switch costs in trial N were substantially smaller after an order switch in trial N – 1 (*M* = 4.0 %) than after an order repetition in trial N – 1 (*M* = 9.0 %). The second set of tests analyzed the specific nature of this reduction in order switch costs. Responses in order repetitions in trial N were significantly more incorrect after a different order (vs. same order) in trial N -1, *t*(22) = 3.956, *p* < .001. At the same time, error rates of different orders in trial N did not differ after a different order (vs. same order) in trial N – 1, *t*(22) = 1.539, *p* = .138.

In Session 4 of the dual-task practice group (Figure 2B), the first set compared same-order and different-order trials in the current trials after different order trials in trial N – 1, *t*(22) = 1.690, *p* = .105, as well as after same-order trials in trial N – 1, *t*(22) = 3.104, *p* < .001. Order switch costs in trial N were substantially smaller after an order switch in trial N – 1 (*M* = 0.9 %) than after an order repetition in trial N – 1 (*M* = 1.3 %). The second set of tests analyzed the specific nature of this reduction in order switch costs. However, error rates in order repetitions in trial N did not differ after a different order (vs. same order) in trial N -1, *t*(22) < 1. Similarly, error rates of different orders in trial N did not differ after a different order (vs. same order) in trial N – 1, *t*(22) < 1.

In Session 4 of the single-task practice group (Figure 2C), the first set compared same-order and different-order trials in the current trials after different order trials in trial N – 1, *t*(22) = 2.800, *p* < .001, as well as after same-order trials in trial N – 1, *t*(22) = 5.410, *p* < .001. Although both tests were significant, order switch costs in trial N were substantially smaller after an order switch in trial N – 1 (*M* = 2.2 %) than after an order repetition in trial N – 1 (*M* = 4.3 %). The second set of tests analyzed the specific nature of this reduction in order switch costs. However, error rates in order repetitions in trial N did not differ after a different order (vs. same order) in trial N -1, *t*(22) = 1.925, *p* = .067. Similarly, error rates of different orders in trial N did not differ after a different order (vs. same order) in trial N – 1, *t*(22) = 1.816, *p* = .083.

**Error2**

In Session 1 of the dual-task practice group (Figure 2D), the first set compared same-order and different-order trials in the current trials after different order trials in trial N – 1, *t*(22) = 3.952, *p* < .001, as well as after same-order trials in trial N – 1, *t*(22) = 6.057, *p* < .001. Although both tests were significant, order switch costs in trial N were substantially smaller after an order switch in trial N – 1 (*M* = 4.1 %) than after an order repetition in trial N – 1 (*M* = 9.8 %). The second set of tests analyzed the specific nature of this reduction in order switch costs. Responses in order repetitions in trial N were significantly more incorrect after a different order (vs. same order) in trial N -1, *t*(22) = 2.812, *p* = .010. At the same time, error rates of different orders in trial N did not differ after a different order (vs. same order) in trial N – 1, *t*(22) = 1.734, *p* = .097.

In Session 4 of the dual-task practice group (Figure 2E), the first set compared same-order and different-order trials in the current trials after different order trials in trial N – 1, *t*(22) < 1, as well as after same-order trials in trial N – 1, *t*(22) = 3.326, *p* = .003. Order switch costs in trial N were substantially smaller after an order switch in trial N – 1 (*M* = 0.1 %) than after an order repetition in trial N – 1 (*M* = 1.7 %). The second set of tests analyzed the specific nature of this reduction in order switch costs. However, error rates in order repetitions in trial N did not differ after a different order (vs. same order) in trial N -1, *t*(22) = 1.916, *p* = .068. Similarly, error rates of different orders in trial N did not differ after a different order (vs. same order) in trial N – 1, *t*(22) < 1.

In Session 4 of the single-task practice group (Figure 2F), the first set compared same-order and different-order trials in the current trials after different order trials in trial N – 1, *t*(22) = 2.134, *p* = .044, as well as after same-order trials in trial N – 1, *t*(22) = 4.454, *p* < .001. Although both tests were significant, order switch costs in trial N were substantially smaller after an order switch in trial N – 1 (*M* = 1.9 %) than after an order repetition in trial N – 1 (*M* = 3.2 %). The second set of tests analyzed the specific nature of this reduction in order switch costs. However, error rates of order repetitions in trial N did not differ after a different order (vs. same order) in trial N -1, *t*(22) < 1. Similarly, error rates of different orders in trial N did not differ after a different order (vs. same order) in trial N – 1, *t*(22) = 1.416, *p* = .171.

**Response reversals**

In Session 1 of the dual-task practice group (Figure 3A), the first set compared same-order and different-order trials in the current trials after different order trials in trial N – 1, *t*(22) = 4.843, *p* < .001, as well as after same-order trials in trial N – 1, *t*(22) = 6.350, *p* < .001. Although both tests were significant, order switch costs in trial N were substantially smaller after an order switch in trial N – 1 (*M* = 3.9 %) than after an order repetition in trial N – 1 (*M* = 9.8 %). The second set of tests analyzed the specific nature of this reduction in order switch costs. Response reversal rates in order repetitions in trial N were significantly increased after a different order (vs. same order) in trial N - 1, *t*(22) = 3.615, *p* = .002. At the same time, response reversal rates of different orders in trial N were significantly decreased after a different order (vs. same order) in trial N - 1, *t*(22) = 2.079, *p* = .049.

In Session 4 of the dual-task practice group (Figure 3B), the first set compared same-order and different-order trials in the current trials after different order trials in trial N – 1, *t*(22) = 1.005, *p* = .326, as well as after same-order trials in trial N – 1, *t*(22) = 3.017, *p* = .006. Order switch costs in trial N were substantially smaller after an order switch in trial N – 1 (*M* = 0.4 %) than after an order repetition in trial N – 1 (*M* = 1.2 %). The second set of tests analyzed the specific nature of this reduction in order switch costs. However, response reversal rates in order repetitions in trial N did not differ after a different order (vs. same order) in trial N -1, *t*(22) = 1.489, *p* = .151. Similarly, response reversal rates of different orders in trial N did not differ after a different order (vs. same order) in trial N – 1, *t*(22) < 1.

In Session 4 of the single-task practice group (Figure 3C), the first set compared same-order and different-order trials in the current trials after different order trials in trial N – 1, *t*(22) = 2.796, *p* = .011, as well as after same-order trials in trial N – 1, *t*(22) = 3.994, *p* < .001. Although both tests were significant, order switch costs in trial N were substantially smaller after an order switch in trial N – 1 (*M* = 2.1 %) than after an order repetition in trial N – 1 (*M* = 3.3 %). The second set of tests analyzed the specific nature of this reduction in order switch costs. However, response reversal rates in order repetitions in trial N did not differ after a different order (vs. same order) in trial N -1, *t*(22) = 1.528, *p* = .141. Similarly, response reversal rates of different orders in trial N did not differ after a different order (vs. same order) in trial N – 1, *t*(22) < 1.

**Pre-test differences** **(see main document for explanations, Table 1)**

**RTs**

The reduced RT1 order switch costs after dual-task practice do not result from performance differences at the beginning of dual-task and single-task practice because, during the pre-test, the RT1 analysis showed no main effect of or interaction with GROUP, *F*s(1, 44) < 1. Similarly, the reduced RT2 costs after dual-task practice also do not result from differences between the dual-task and single-task practice groups at the beginning of practice because, during the pre-test, the RT2 analysis showed no main effect of or interaction with GROUP, *F*s(1, 44) < 1.389, *p*s > .252.

**Errors**

The reduced Error1 order switch costs after dual-task practice do not result from differences before practice, because the Error1 analysis showed no main effect of or interaction with GROUP during the pre-test, *F*s(1, 44) < 2.358, *p*s > .131. Similarly, the reduced Error2 costs after dual-task practice do not result from differences at the beginning of practice because the Error2 analysis showed no main effect of or interaction with GROUP during the pre-test, *F*s(1, 44) < 1.029, *p*s > .324.

**Response reversal rates**

The reduced reversal rate costs after dual-task practice do not result from differences at the beginning of practice because the analysis of the reversal rates during the pre-test showed no main effect of GROUP, *F*(1, 44) = 3.459, *p* = .066, or an interaction of CURRENT ORDER and GROUP, *F*(1, 44) < 1.

**Table 1.** Pre-test data for Reaction time on Task 1 (RT1) in ms, Reaction time on Task 2 (RT2) in ms, error rates on Task 1 (Error1) in %, error rates on Task 2 (Error2) in %, and the reversal rates in % in the dual-task practice group and the single-task practice group.

| **Group** | **CURRENT ORDER condition** | **RT1** | **RT2** | **Error1** | **Error2** | **Reversal rate** |
| --- | --- | --- | --- | --- | --- | --- |
| **Dual-task practice group** | **Same order** | 1,180 (51) | 1,183 (49) | 14.4 (3.7) | 16.8 (3.4) | 11.4 (2.8) |
|  | **Different order** | 1,376 (57) | 1,391 (60) | 23.3 (3.7) | 26.2 (3.9) | 20.0 (3.2) |
| **Single-task practice group** | **Same order** | 1,239 (81) | 1,246 (87) | 9.9 (2.1) | 14.6 (2.9) | 6.5 (1.6) |
|  | **Different order** | 1,394 (85) | 1,395 (91) | 15.6 (1.9) | 21.3(2.5) | 13.0 (1.6) |

**Practice effects within Session 4 following dual-task and single-task practice (see main document for explanations)**

**RTs**

The reduced RT1 order switch costs after dual-task practice were not significantly modulated by practice within Session 4. This conclusion resulted from a block-wise analysis of RT1, showing no significant interaction of BLOCK (Block 1 to 10), CURRENT ORDER, and GROUP, *F*(9, 396) = 1.575, *p* = .120. Similarly, these reduced RT2 costs after dual-task practice were not significantly modulated by practice within Session 4. This conclusion resulted from a block-wise analysis of RT2, showing no significant interaction of BLOCK (Block 1 to 10), CURRENT ORDER, and GROUP, *F*(9, 396) = 1.557, *p* = .126.

**Errors**

The reduced Error1 order switch costs after dual-task practice were not significantly modulated by practice within Session 4. This conclusion resulted from a block-wise analysis of Error1, showing no significant interaction of BLOCK (Block 1 to 10), CURRENT ORDER, and GROUP, *F*(9, 396) = 1.059, *p* = .392. Similarly, these reduced Error2 costs after dual-task practice were not significantly modulated by practice within Session 4. This conclusion resulted from a block-wise analysis of Error2, showing no significant interaction of BLOCK (Block 1 to 10), CURRENT ORDER, and GROUP, *F*(9, 396) = 1.619, *p* = .108.

**Response reversal rates**

Different to all previous analyses, the block-wise analysis of the response reversal rates in Session 4 showed a significant interaction of BLOCK (Block 1 to 10), CURRENT ORDER, and GROUP, *F*(9, 396) = 1.966, *p* = .042, *ŋp²* = .04. However, this interaction does no clearly point to a reduction of the group differences regarding the order switch costs with practice during Session 4, because these costs developed with no systematic pattern in the dual-task practice group (Block 1: -0.7 %, Block 2: 1.4 %, Block 3: 1.1 %, Block 4: 2.1 %*, Block 5: 2.0 %*, Block 6: 2.3 %*, Block 7: -0.5 %, Block 8: 1.2 %, Block 9: 0.6 %, Block 10: -0.1 %; values with an asterisk * represent significant costs) and the single-task group (Block 1: 4.3 %*, Block 2: 3.6 %*, Block 3: 1.5 %, Block 4: 2.4 %*, Block 5: 1.1 %, Block 6: 0.9 %, Block 7: 3.0 %*, Block 8: 3.2 %*, Block 9: 3.8 %*, Block 10: 3.1 %*).
